# Source-to-sink transport of sugar and regulation by environmental factors

**DOI:** 10.3389/fpls.2013.00272

**Published:** 2013-07-24

**Authors:** Remi Lemoine, Sylvain La Camera, Rossitza Atanassova, Fabienne Dédaldéchamp, Thierry Allario, Nathalie Pourtau, Jean-Louis Bonnemain, Maryse Laloi, Pierre Coutos-Thévenot, Laurence Maurousset, Mireille Faucher, Christine Girousse, Pauline Lemonnier, Jonathan Parrilla, Mickael Durand

**Affiliations:** ^1^Unités Mixtes de Recherche, Ecologie et Biologie des Interactions, Université of Poitiers/Centre National de la Recherche ScientifiquePoitiers, France; ^2^Diversité et Ecophysiologie des Céréales, Unités Mixtes de RechercheClermont Ferrand, France

**Keywords:** Phloem, sugar transport, source/sink, abiotic factors, biotic factors

## Abstract

Source-to-sink transport of sugar is one of the major determinants of plant growth and relies on the efficient and controlled distribution of sucrose (and some other sugars such as raffinose and polyols) across plant organs through the phloem. However, sugar transport through the phloem can be affected by many environmental factors that alter source/sink relationships. In this paper, we summarize current knowledge about the phloem transport mechanisms and review the effects of several abiotic (water and salt stress, mineral deficiency, CO_2_, light, temperature, air, and soil pollutants) and biotic (mutualistic and pathogenic microbes, viruses, aphids, and parasitic plants) factors. Concerning abiotic constraints, alteration of the distribution of sugar among sinks is often reported, with some sinks as roots favored in case of mineral deficiency. Many of these constraints impair the transport function of the phloem but the exact mechanisms are far from being completely known. Phloem integrity can be disrupted (e.g., by callose deposition) and under certain conditions, phloem transport is affected, earlier than photosynthesis. Photosynthesis inhibition could result from the increase in sugar concentration due to phloem transport decrease. Biotic interactions (aphids, fungi, viruses…) also affect crop plant productivity. Recent breakthroughs have identified some of the sugar transporters involved in these interactions on the host and pathogen sides. The different data are discussed in relation to the phloem transport pathways. When possible, the link with current knowledge on the pathways at the molecular level will be highlighted.

## SUGAR TRANSPORT IN THE PHLOEM

Among the sugars synthesized in a plant, only a few are transported in the phloem over a long-distance, whatever the species and the type of phloem loading considered. In all cases, sucrose is the main form of carbon found in the phloem. In addition to sucrose, polyols (mainly sorbitol and mannitol) and oligosaccharides of the raffinose family can also be found. In some species, both polyols and raffinose are found in the phloem (Rennie and Turgeon, 2009). Hexose transport in the phloem has also been reported for a limited number of species (van Bel and Hess, 2008) but these results were recently challenged (Liu et al., 2012). Raffinose and other members of the raffinose family oligosaccharides are indirectly involved in the building up of sugar concentrations in the phloem by polymer trapping (Rennie and Turgeon, 2009). Conversely, polyols tend to behave exactly like sucrose as far as transport is concerned and thus, in apoplastic loaders, there are specific polyol transporters (Noiraud et al., 2001b). Unless stated otherwise, sucrose is the main sugar we deal with in the following sections.

**FIGURE 1:**
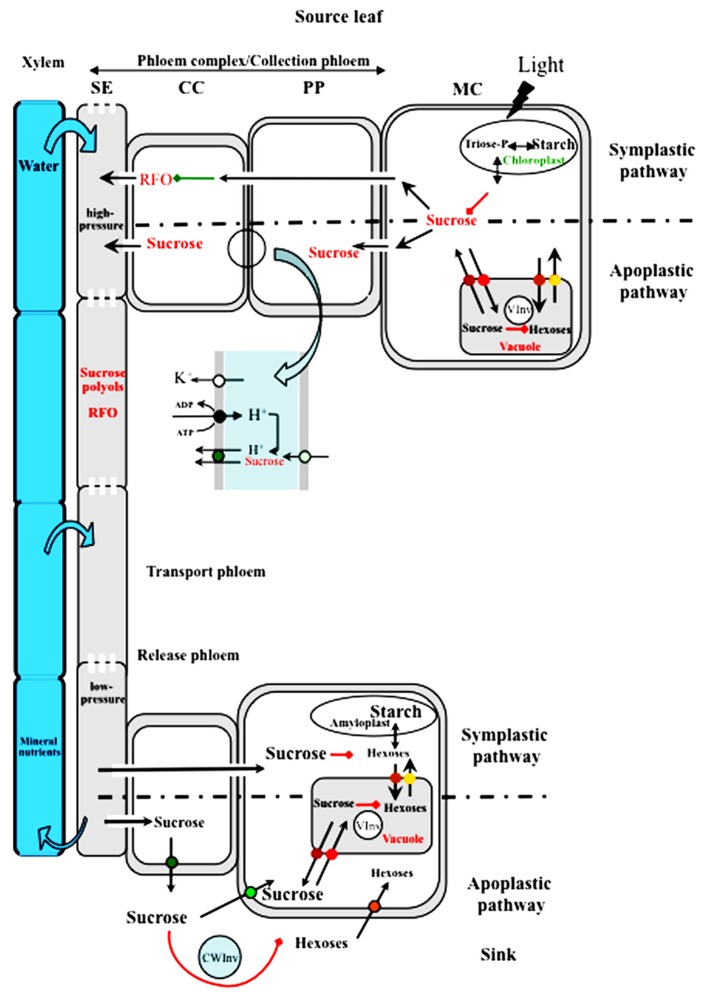
**Comparison of source-to-sink sugar transport in symplastic and apoplastic active phloem loaders.** Sucrose available for export from mesophyll cells (MC) results from a balance between storage in the vacuoles and sequestration as starch in the chloroplasts. Sucrose can reach the sieve tubes through plasmodesmata that allow for its diffusion from cell to cell in species like cucurbits. Sucrose is converted to larger molecules (RFOs) by the sequential addition of galactosyl residues in modified companion cells (CC) called intermediary cells. The larger molecules cannot move back to phloem parenchyma cells (PP) and are transferred and accumulated in sieve tubes. In apoplast-loading species, sucrose reaches phloem parenchyma cells through plasmodesmata. Sucrose is loaded and accumulates in the phloem by passing through the apoplast between the PP and the CC. The major players are presented in the enlargement of that area. Sucrose enters the apoplast through facilitators of the SWEET family (pale green circle) and is accumulated in the companion cell by a proton/sucrose co-transporter of the SUT1/SUC2 type (green circle). The energy necessary for the co-transport is provided by an H+/pumping ATPase (black circle) which establishes a proton gradient and a trans-membrane potential regulated by potassium channels of the AKT2/3 type (white circle). In Solanaceous species, SUT1 transporters are localized at the plasma membrane of sieve elements (not shown). Polyols can also be transported into the phloem, with specific transporters located in the plasma membrane of CC (not shown). A high hydrostatic pressure is generated in the sieve tubes of the collection phloem and water from the xylem is attracted. Sucrose, RFOs and polyols are transported in the sieve tubes to the sink organs in the transport phloem. All along the path, they can be leaked from and reloaded into the phloem *via* the same mechanism (not shown). Sucrose is unloaded into the release phloem where the hydrostatic pressure is supposed to be lower. Sucrose can be unloaded through a symplastic pathway or through an apoplastic pathway. In the latter case, sucrose is unloaded into the apoplast through specific carriers which can be of the SUT1/SUC2 type (green circle; Carpaneto et al., 2005). Sucrose is then taken up by sink-specific sucrose carriers of the same SUT1/SUC2 (light green circle) or converted to hexoses by a cell-wall invertase (CWInv). Hexoses are then taken up by specific carriers at the plasma membrane (orange circle) or at the tonoplast level (yellow and brown circles). Sucrose in sink cells can be metabolized (growing sinks) or stored as starch in amyloplasts, or imported into the vacuoles (red circles) and further converted to hexoses by a vacuolar invertase (VInv).

According to many studies, up to 80% of photosynthetic fixed carbon can be exported by mature leaves. The amount of sucrose available for export from source leaves depends on several parameters: photosynthetic activity (carbon fixation), partitioning between starch synthesis in the chloroplast and triose-phosphates exported from the chloroplast for sucrose synthesis, and transient storage of sucrose in the vacuole (**Figure 1**). If one of these factors is altered, the amount of sucrose available for export is affected and therefore source/sink relationships can be altered. The pathways for sucrose loading in the conducting cells of the phloem have been documented mostly in the case of active phloem loading in herbaceous species (**Figure 1**). Active phloem loading results in a higher solute concentration in the sieve element-companion cell complex (SE/CC complex) than in the surrounding tissues. The mechanism of active phloem loading from the apoplastic space involves sucrose and polyol transporters that have been identified in numerous species (Noiraud et al., 2001b; Lalonde et al., 2004; Sauer, 2007; Reinders et al., 2012). These transporters can concentrate sugars in the SE/CC complex by dissipating the proton gradient established by an H^+^/ATPase located in the same cells. The release of sucrose in the apoplast in the vicinity of the SE/CC complex may be controlled by the recently discovered SWEET facilitators (Chen et al., 2012; **Figure 1**). The second mechanism for active phloem loading is polymer trapping, whereby sucrose is converted to raffinose or larger molecules through addition of galactose to sucrose in intermediary cells (Rennie and Turgeon, 2009). In that case, sugars move from cell to cell through a symplastic pathway (**Figure 1**). Active phloem loading may not be universal as there are many indications of passive loading at least in tree species (Rennie and Turgeon, 2009; Turgeon, 2010b). This is achieved by maintaining high solute concentrations in the mesophyll cells of such species.

These different pathways concern the loading of sucrose in the so-called collection phloem (Van Bel, 2003) which represents the initial step of long-distance transport. Transport along the path between source and sink occurs in the transport phloem and sucrose is delivered to sink organs by the release phloem (Van Bel, 2003). The most widely accepted concept to explain solute transport in the phloem is mass-flow, as initially proposed by Münch and followers, whereby the hydrostatic pressure difference in the phloem between source (high pressure) and sink (low pressure) accounts for sap movement (**Figure 1**). At many stages along the pathway, specific transporters are involved in the cell-to-cell movement of sucrose or in the intracellular compartmentation between the cytoplasm and organelles; they thus represent major regulators of sugar fluxes. It should be noted that sucrose transporters (SUTs) have been localized and characterized in the three phloem sections. Sucrose can act as a signal and regulate many genes involved in growth and development (Koch, 2004; Muller et al., 2011).

During longitudinal transport, sucrose can be leaked and retrieved but also used by sink cells along the path (axial sinks; Minchin and Thorpe, 1987). In some species, stems or petioles can be turned into storage organs (e.g., celery; Noiraud et al., 2001a) and this function is even more pronounced in tree trunks (Hou, 1985). In such conditions, storage is transient as resources will later be used to support growth along with plant development. These organs successively act as sinks and sources (Juchaux-Cachau et al., 2007). Concerning their ability to retrieve sucrose from the apoplast, the respective membrane potential levels between SEs and phloem parenchyma cells are decisive (Hafke et al., 2005). SUTs are involved in sucrose movement in the transport phloem, even in tree species where loading is symplastic in the collection phloem (Turgeon, 2010b).

In the release phloem, sugars can exit the phloem through either a symplastic or an apoplastic pathway, although the first steps are often symplastic (Fisher and Oparka, 1996; Patrick, 1997). However, unloading pathways depend on the particular sink involved and its development stage (**Figure 1**). In sinks like developing seeds or infected tissues, symplastic discontinuity requires an apoplastic step for the transfer of photo-assimilates. A switch from apoplastic to symplastic unloading was noted during potato tuberization (Viola et al., 2001). In fruit development, contrasting results have been found: in grape berry, Zhang et al. (2006) demonstrated a shift from symplastic to apoplastic unloading whereas in apple and cherry fruit there is evidence for an apoplastic step in sucrose and sorbitol unloading, involving transporters (Gao et al., 2003; Zhang et al., 2004).

In seeds, SUTs but also hexose transporters and cell-wall invertases are responsible for sugar movement but their respective roles differ depending on the development stage (Weber et al., 1997; Weschke et al., 2003). These pathways have been extensively studied in legume seeds, together with the corresponding regulation of sucrose unloading (Zhang et al., 2007).

## SOURCE-SINK RELATIONSHIPS IN PLANT AND SUGAR ALLOCATION (SINK STRENGTH)

Sink organs depend on the delivery of sucrose (or other forms of carbohydrates) by the phloem for their growth and development. A plant may be regarded as a series of sources and sinks with an overall carbon fixation capacity and several sinks “ competing” for the available photo-assimilates. This creates a priority system among sinks. Roots and young leaves are major sinks during the early developmental stages, whereas tubers, fruit and seeds become major sinks during the reproductive stages (Wardlaw, 1990). The distribution of resources among sinks is also a key factor of plant productivity based on the harvest index (HI). The HI is the ratio of harvested dry weight over plant dry weight (or above-ground shoot dry weight): therefore a high HI indicates that a large amount of photo-assimilates has been diverted to the sinks harvested by humans (Gifford et al., 1984).

In order for plants to reach a balanced development and optimize their reproductive fitness, priority for access to photo-assimilates needs to be established between sinks. Changes in carbon partitioning and switches between the apoplastic and symplastic pathways occur throughout development or as a response to the environment (Roitsch, 1999; Godt and Roitsch, 2006). Concerning the apoplastic pathway, hexose transport resulting from cell-wall invertase/hexose transporter activity has been suggested to predominate in the sink tissues that undergo cell division and elongation, while sucrose transport predominates in the sink tissues that switch to storage mode (Weschke et al., 2000, 2003).

Priority among sinks has been related to the so-called “ sink strength” (Ho, 1988) or sink dominance. According to Wardlaw (1990), the underlying basis of sink strength (assuming a pressure flow mechanism for translocation) is an ability to effectively lower the concentration of photo-assimilates in the SEs of the sinks and thus establish a favorable hydrostatic pressure gradient between the source and the sink. In that respect, the role of cell-wall invertases has frequently been highlighted in sink organs as they increase sucrose unloading by converting sucrose to hexoses. Transport of photo-assimilates depends on source supply and sink demand. The role of the phloem sap sugar content in the coupling between sink demand and source activity is still a matter of debate (Minchin et al., 2002). However, high sucrose contents in leaves could have an inhibitory effect on SUT activity and thus inhibit sucrose loading into the phloem. This point was evidenced by feeding sucrose to the transpiration stream of cut sugar-beet leaves (Chiou and Bush, 1998). The authors hypothesized that high sucrose concentrations in the vascular tissue, resulting from decreased sink demand, down-regulated transporter activity. This could lead to decreased phloem loading and increased sugar levels in mesophyll cells, and in turn down-regulated photosynthesis. The opposite regulation is thought to occur in the case of increased sink demand (Chiou and Bush, 1998). This link between sugar export in leaves and sink demand has been re-examined by Ainsworth and Bush (2011) and phloem sucrose transport has been identified as a possible target for improving plant productivity.

Phloem transport capacity may not be a limiting factor, as shown in several reports. In transgenic sugarcane, expressing a sucrose isomerase led to the accumulation of sucralose in addition to sucrose in stalk vacuoles. The sugar concentration was therefore doubled in the juice harvested from stalks (Wu and Birch, 2007). In such plants, photosynthesis and sucrose transport were greatly increased, indicating a release of sink limitation. The overexpression of an *Arabidopsis* tonoplastic glucose transporter (*TMT1*) led to increased glucose contents in the vacuoles of mesophyll cells and to higher seed yield. In these plants, higher expression levels of *AtSUC2*, the transporter that loads sucrose into the phloem in *Arabidopsis*, have been noted (Wingenter et al., 2010). However, TMT1 can also drive sucrose entry into the vacuole (Schulz et al., 2011) and therefore the former interpretation may have to be reconsidered. In rice, when the expression of a SUTs involved in sucrose efflux from the vacuole (OsSUT2) was suppressed, seed production as well as root growth were reduced, indicating that sucrose transport to sinks was impaired (Eom et al., 2011) and confirming the former results obtained in *Arabidopsis*. Altogether these different data suggest a link between the sugar concentration in the cytoplasm of mesophyll cells and the export of sucrose, and indicates source-limitation in wild-type plants.

Taking the former elements into account, source-to-sink sucrose transport can be affected by environmental factors at least at three different levels (**Figure 1**):

(i) the source (e.g., by an effect on photosynthesis or phloem loading), resulting in less sucrose available for export,
(ii) the sink (e.g., increased demand for root growth, pathogens developing on plant organs), leading to a new balance between sinks that can be detrimental to yield,
(iii) the path between source and sink (by e.g., cold treatment, aphids, viruses) leading to impaired sucrose delivery.

Plants undergo large changes in their environment throughout their life and have developed many strategies to respond to these changes. The following sections will try to summarize some of the effects of environmental factors on sucrose transport from source to sink organs.

## EFFECTS OF ABIOTIC FACTORS

Among the many environmental factors that can affect plant growth, the present review concentrates on two types: environmental cues and some air and soil pollutants.

### EFFECTS OF ENVIRONMENTAL CUES

#### Effects of water deficit

Water deficit is a major abiotic factor affecting crop development and yield. Drought imposes unfavorable conditions on the leaves (source) and roots (sink) of a plant. However, as pointed out by Turgeon (2010a), the high osmotic potential in the phloem can be a positive parameter for attracting water to the sieve tubes and maintaining phloem sap flow in drought conditions.

Under mild water deficit, shoot growth is restricted while root growth continues and, consequently, plant architecture is modified. In dicots, e.g., pea and grape, the number of branches and the number of leaves on branches are particularly sensitive to soil water deficit (Lecoeur et al., 1995; Lebon et al., 2006). In monocots such as grasses, the number of young emerging organs is reduced under drought (Courtois et al., 2000). As a result of this water-stress avoidance strategy, global photosynthetic productivity may decrease and thus impact the carbon flow to different sink organs.

Most research on the effect of water deficit on sugar metabolism and phloem loading has been led using sucrose-translocating species and demonstrates that in leaves, carbohydrate levels are altered by drought. Sucrose and hexose amounts increase, while starch levels decrease (Pelleschi et al., 1997), suggesting the induction of starch hydrolysis and sucrose synthesis. In cotton, water-stress-induced accumulation of sucrose in the source leaves has been hypothesized as providing an energy supply to maintain cell survival in high-respiration environments (Burke, 2007). Furthermore, sucrose and hexose accumulation is considered to play a major role in osmotic adjustment to maintain metabolic activity in source leaves. However, sugars may also accumulate in leaves because of a decreased demand as a consequence of growth limitation (Hummel et al., 2010).

The effects of water deficit on species that translocate raffinose family oligosaccharides (RFOs) were also investigated since RFOs are involved in desiccation tolerance in seeds (Koster and Leopold, 1988) and in low-temperature acclimation of leaves (Bachmann and Keller, 1995). A hypothetical model depicting the effects of water-deficit stress on the carbon flow between RFOs and *O*-methyl-inositol (OMI) has been proposed in *Coleus*, a drought-tolerant plant. Under stress, reduced RFO levels were observed and, conversely, increased OMI synthesis was noted. The two metabolic pathways share myo-inositol, a ubiquitous plant cyclitol, as an intermediate. In *Coleus*, the activity of galactinol synthase, an enzyme that catalyses the first step of RFO biosynthesis from UDP-galactose, was found down-regulated by water deficit, thus contributing to lower levels of transportable RFOs (Pattanagul and Madore, 1999).

In source leaves, transcript abundance of several genes encoding enzymes involved in gluconeogenesis such as fructose-biphosphate aldolase (Cramer et al., 2007), in the phosphorylation of soluble sugars such as hexokinase (Whittaker et al., 2001), and in RFO biosynthesis such as galactinol synthase (Taji et al., 2002) increased in response to dehydration stress. Transgenic *Arabidopsis* plants that overexpressed galactinol synthase produced elevated amounts of galactinol and raffinose, which may function as osmoprotectants and contribute to water-deficit stress tolerance (Taji et al., 2002). Such an apparent discrepancy between RFO-transporting plants such as *Coleus* and sucrose-transporting ones, such as *Arabidopsis*, may reflect differential responses of distinct species to water stress.

Water deficit induces changes in the concentrations of the main organic nutrients that move inside the sieve tubes, i.e., sugars and amino acids. Analysis of alfalfa phloem sap, collected by stylectomy, indicated a significant increase in sucrose contents and total amino acid concentrations as the leaf water potential decreased from -0.4 to -2.0 MPa. The change in total amino acid concentration was due to larger amounts of Val, Leu, Ile, Glu, Asp, Thr, and especially Pro (Girousse et al., 1996). Similarly, water stress induced increased sucrose and Pro levels in phloem sap collected from cut petioles of *Arabidopsis *leaves by the EDTA method (Mewis et al., 2012).

In sink organs, examples of the negative effects of drought on sink growth have been reported in potato tubers, where osmotic stress promoted sucrose biosynthesis instead of starch biosynthesis *via* the induction of sucrose-phosphate synthase (SPS) and the inhibition of ADP glucose pyrophosphorylase (AGPase; Geigenberger et al., 1997). The degradation of some storage carbohydrates such as starch and fructans in stems has been correlated to starch accumulation in grains (Yang et al., 2004). Likewise, drought led to a fivefold decrease in cytosolic invertase activity in the seeds of *Lupinus albus* (Pinheiro et al., 2005), together with an increase in the activity of vacuolar and cytosolic invertases in leaves, suggesting that the amounts of sucrose available for transport to the seeds are reduced under drought (Kim et al., 2000; Pinheiro et al., 2001; Trouverie et al., 2004). As a conclusion, several observations bring evidence for negative effects of dehydration on the sink/source ratio that are detrimental to crop production in terms of biomass redistribution (Cuellar-Ortiz et al., 2008).

Data about the involvement of SUTs in drought and salinity tolerance remain limited. Group IV SUTs have been identified as tonoplast-localized SUT/H+ symporters able to regulate sucrose movement from the vacuole to the cytosol. In rice photosynthetic leaves, the SUT OsSUT2 was up-regulated during drought and salinity treatments (Ibraheem et al., 2011). Using *PtaSUT4*-RNAi-suppressed poplars, (Frost et al., 2012) demonstrated the effects of altered sucrose compartmentation on photosynthesis efficiency and accumulation of water-stress-related RFOs in source leaves. The authors suggest that export and long-distance sucrose transport may be at least partly controlled by SUT-mediated sucrose sequestration within the vacuole.

The effects of water deficit have also been studied at different development stages. Drought stress can induce senescence and enhance reserve mobilization (Chandlee, 2001). In other terms, senescence and reserve mobilization are integral components of plant development and basic strategies in stress mitigation (Cowan et al., 2005). Studies using tomato plants over-expressing *Arabidopsis* hexokinase showed that increased hexokinase levels in plants induced higher sugar contents, which reduced photosynthetic activity and consequently accelerated senescence in leaves (Dai et al., 1999). Sugar levels can influence leaf progress through senescence as direct causal signals, but also as substrates for carbon mobilization and reallocation to allow plants to alleviate the effects of drought stress (Wingler et al., 1998; Rizhsky et al., 2004; Cramer et al., 2007).

In rice, drought-induced leaf senescence promotes allocation of assimilates to developing grains, shortens grain filling, and increases the grain filling rate (Yang et al., 2002). In soybean, water depletion decreases seed size primarily because of a shortening of the filling period rather than an inhibition of the seed growth rate (Westgate et al., 1989). Since seed growth depends on the supply of assimilates from the maternal plant (source activity), as well as on the demand for assimilates within the embryonic tissues (sink activity), both maternal and embryonic factors contribute to the maintenance of seed growth under water deficit. Thus, the latter authors hypothesized that a rapid depletion of sucrose in and around the embryo would point to a source limitation, whereas a reduction in sucrose uptake would imply a sink limitation. Even though severe water deficit completely inhibits photosynthesis and decreases the sucrose concentration in the cotyledon apoplast by approximately 50%, seeds continue to accumulate dry matter at or near the control rate. Reserve carbohydrates are thus mobilized from all source organs (leaves, stems, and pericarp tissue), and this enhances the apoplastic and/or symplastic supply to support seed filling; water-deficient plants display an increased rate of sucrose uptake relative to their well-watered controls (Westgate et al., 1989), consistent with source limitation.

As an example of fruit development, the ripening grape berry represents a well-characterized example of a very strong sugar sink. Grape yield is reduced under drought, while total sugar content in the surviving berries increases (Huglin, 1986). The early development of grape berry appears as the most drought-sensitive stage, but in spite of negative effects on berry growth, drought does not affect sugar accumulation, confirming that sink strength within individual berries is set by sink activity, not by berry size, as reviewed by Agasse et al. (2009). A shift from a symplastic- to an apoplastic-unloading pathway has been demonstrated. It occurs at the onset of ripening, and is accompanied by a concomitant increase of the expression and activity of cell-wall invertases, leading to a massive import of hexoses (Zhang et al., 2006).

Altogether these data indicate that sensitivity to water deficit is particularly acute during reproductive development because photo-assimilate allocation to newly established sinks such as flowers, seeds, and fruit, can be compromised by competition with roots under drought stress. In order to apply this knowledge to crop improvement, more detailed understanding of drought sensitivity at that crucial stage for productivity is needed. In that respect, it is no surprise that selection for drought resistance should result in the choice of traits affected by modifications in the sink/source relationship in response to drought, such as the accumulation of biomass in reproductive organs (Schnyder, 1993).

#### Effects of mineral deficiency

Plants acquire mineral nutrients for their growth and development through the roots. Plasticity of the root system architecture is therefore a key adaptation feature that allows plants to cope with a changing environment. As pointed out by Hermans et al. (2006), plants generally respond to a shortage in mineral nutrition by allocating more resources to the organs involved in mineral acquisition, and this results in a larger root surface. Therefore any depletion in mineral supply can have dramatic effects on resource allocation in plants. Marschner et al. (1996) proposed that nutrient deficiency can affect photo-assimilate partitioning either directly *via *phloem loading and transport or indirectly by depressing sink demand.

As a consequence of plant growth reduction or inhibition by mineral deficiency, sugar concentrations increase in plants and in phloem sap (Peuke, 2010). The question remains as to whether the phloem sugar concentration is a stress response and/or a stress signal (Peuke, 2010).

**FIGURE 2:**
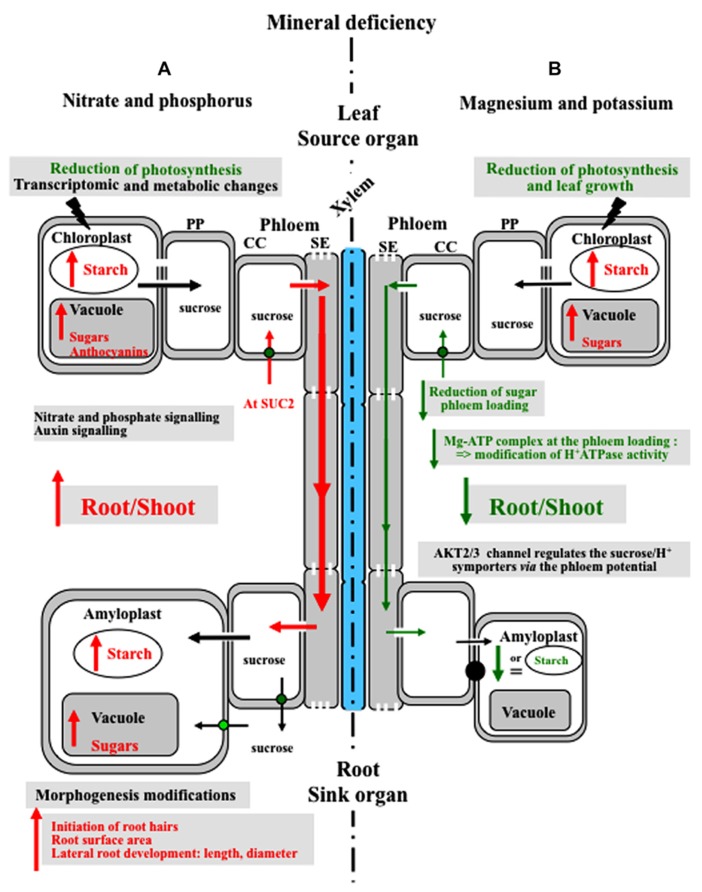
**Model of the plant’s responses to mineral nutrient deficiency.** **(A)** Response to nitrate and phosphorus deficiency: deficiency in nitrogen and phosphorus leads to reduced photosynthesis, accumulation of sugars in source leaves, increased carbon allocation to the roots and a higher root/shoot ratio. Moreover, phosphorus limitation induces an adaptation of the root system architecture: root hairs initiate and elongate, which increases the root surface area. AtSUC2 (green circle) is a component of the sugar-signaling pathway in the response to phosphorus starvation. **(B)** Response to magnesium and potassium deficiency: Mg deficiency increases the concentration of soluble sugars and starch in leaves and reduces leaf growth. Mg deficiency impacts sugar metabolism, as well as sucrose export to the roots. Mg deficiency reduces the Mg-ATP availability and the activity of H^+^-ATPase, thus reducing the driving force for sucrose phloem loading. AKT2/3 potassium channels affect sugar loading and long-distance transport by regulating the H^+^^/^sucrose transporter. Conversely, K^+^-limitation rarely results in starch accumulation. MC, mesophyll cell; CC, companion cell; PP, parenchyma phloem; MC, mesophyll cell.

**Response to nitrate limitation.** Deficiency in nitrogen leads to an accumulation of carbohydrates in leaves and to a higher level of carbon allocated to the root (**Figure 2**) that increases the root/shoot ratio (Marschner et al., 1996; Scheible et al., 1997; Remans et al., 2006). Scheible et al. (1997) reported that the root growth rate is correlated with the root sugar content, and nitrate accumulation in the shoot acts as a signal to regulate root/shoot allocation in tobacco. Sugars accumulated in the leaves of N-deficient plants lead to reduced photosynthesis probably due to feedback metabolite regulation (Martin et al., 2002; **Figure 2**). Nitrogen deficiency reduces photosynthesis by a decrease in RubisCO amount and activity and also a decrease in electron transfer (Paul and Driscoll, 1997; Antal et al., 2010). Hermans et al. (2006) gave some clues as to how N deficiency alters carbohydrate metabolism in the shoot and increases the root/shoot biomass ratio. *Arabidopsis* microarray data suggest that genes related to primary metabolism and carbohydrate metabolism such as starch metabolism, glycolysis, and disaccharide metabolism are significantly over-represented among the differentially regulated genes in the shoots of N-deficient plants (Hermans et al., 2006). All these data show that nitrogen affects the distribution of sugars across plant organs.

**Response to phosphorus limitation.** Phosphorus is the second most limiting mineral nutrient for crop production after nitrogen. Lack of phosphorus in leaf mesophyll cells has a direct effect on photosynthesis through Pi availability in the chloroplast and leads to reduced carbon assimilation (**Figure 2**). Nevertheless, sucrose translocation into the phloem is maintained and sometimes increased at least during the early phases of phosphorus starvation (up to 6 days; Hermans et al., 2006).

Like N deficiency, phosphorus limitation induces increased photo-assimilate allocation to the roots and an adaptation of the root system architecture. Root hairs initiate and elongate in response to phosphate starvation, increasing the root surface area (Hammond and White, 2008).

The importance of phloem sucrose transport in P-deficiency signaling has been clearly demonstrated by Liu et al. (2005). In white lupin roots, two genes responsible for phosphate acquisition (*LaPTI*, a phosphate transporter and *LaSAP1*, a secreted acid phosphatase) are rapidly induced by phosphate starvation. When phosphate-starved plants were treated by phloem girdling to prevent shoot-to-root sucrose transport, no induction of either *LaPT1* or *LaSAP1* was noted. In such conditions, delivery to the roots of ^14^C-sucrose applied onto leaflets was reduced by 95% in stem-girdled plants. Sucrose transport from shoot to root was therefore necessary for phosphate starvation signaling.

In a search for *Arabidopsis* plants affected in secreted acid phosphatase activity, Zakhleniuk et al. (2001) identified *pho3*, a mutant that displays a number of features usually associated with Pi-deficient plants (low Pi, sugar, anthocyanin and starch accumulation in leaves). Moreover, *pho3 *mutants were unable to respond further to low Pi (Zakhleniuk et al., 2001; Lloyd and Zakhleniuk, 2004). The mutation was subsequently located in the At*SUC2* gene (Lloyd and Zakhleniuk, 2004) and a link was thus clearly established between sucrose availability for long-distance transport and the response to P starvation. This was confirmed by the over-expression of AtSUC2 in *Arabidopsis* plants that displayed higher sensitivity to P starvation (Lei et al., 2011; **Figure 2**). Altogether, these data clearly demonstrate that sucrose transport to the root is a necessary signal for the response to phosphate starvation, although recent data identified miRNAs translocated into the phloem as key players in the regulation of mineral nutrition (Kehr, 2013).

**Response to magnesium and potassium deficiency.** Metabolic processes and reactions that are influenced by Mg include chlorophyll formation, photosynthetic carbon dioxide fixation, photo-assimilate phloem loading and partitioning (Cakmak and Yazici, 2010). Accumulation of carbohydrates in leaves is a common phenomenon in Mg-deficient plants (**Figure 2**). Mg deficiency reduces leaf growth more than root growth (**Figure 2**) and impacts on sucrose export to the roots (Hermans et al., 2004; Hermans et al., 2005; Ding, 2011). Mg deficiency is thought to affect phloem sucrose loading by decreasing Mg-ATP availability. Consequently, H^+^-ATPase activity could be inhibited, reducing the driving force for active sucrose phloem loading (Marschner et al., 1996; see **Figure 1**). Moreover, in response to Mg deficiency, the expression**of* BvSUT1*, a gene encoding a companion cell sucrose/H^+^ symporter, was induced in the uppermost expanded leaves, but without further enhancement of sucrose loading into the phloem (Hermans et al., 2005).

K^+^ is the major cation in the phloem. Therefore, changes in its amounts can have dramatic effects on phloem functions. The high sugar concentration measured in the leaves of K-deficient plants does not promote any increase in root sugar content or growth. Deeken et al. (2002) demonstrated that AKT2/3 potassium channel affected sugar loading and long-distance transport by regulating sucrose/H^+^ symporter activity through the pmf (see **Figure 1**). As a consequence, in the *akt2/3* mutants, the amount of sucrose in the phloem sap was drastically reduced. Unlike N deficiency, K^+^ limitation rarely results in starch accumulation.

In conclusion, enhanced carbohydrate transport to the roots has been demonstrated for N and Pi limitation, but not for K or Mg deficiency (Peuke et al., 1994; Hermans et al., 2006; **Figure 2**) and the underlying mechanisms have been partly unraveled.

### EFFECTS OF NaCl

Salt stress, due in many places to irrigation with poor quality water, is considered as a major factor limiting plant growth and productivity. Salt stress shares many features with drought stress because in both cases, the primary effect is a lower soil water potential around the roots. Sodium toxicity, due to transport inside the plant *via* the transpiration stream, adds to that initial stress.

Potassium channels are implied in the recirculation of Na^+^ inside the plant (Berthomieu et al., 2003). Na^+^ can be loaded into the leaf phloem to be directed to roots for excretion, therefore reducing the amount of Na^+^ in leaves (Berthomieu et al., 2003), although that flux may be marginal compared to the xylem flux (Davenport et al., 2007).

Little is known about the effects of salt stress on sucrose translocation into the phloem. Salt stress has an inhibitory effect on photosynthesis (Suwa et al., 2008) and in many cases it leads to growth impairment, more important in leaves than in roots (Lohaus et al., 2000). In maize, phloem sucrose concentrations were not altered by salt stress, whereas amino-acid and Na^+^ contents of the sieve tube sap increased. The higher amount of amino acids delivered to the roots could partly explain the increased root/shoot ratio (Lohaus et al., 2000). However, in tomato, salt stress can have a direct inhibitory effect on phloem sucrose loading and translocation, leading to a deficit in sucrose partitioning to the roots (Suwa et al., 2008).

Resistance to salt stress is frequently associated with polyol-synthesizing plants as polyols are thought to act both as osmotically active and anti-oxydant molecules. When such plants are subjected to salt stress, their polyol content increases in different organs. Polyols are considered as major molecules for plants to cope with stress (Stoop et al., 1996). In polyol-transporting plants, increased polyol synthesis occurs together with an increased expression of genes encoding polyol transporters located in the phloem in Plantago (Pommerrenig et al., 2007), celery (Landouar-Arsivaud et al., 2011) and olive (Conde et al., 2011), suggesting that long-distance polyol transport is also enhanced in response to salt stress. Increased delivery of polyols to roots could have a positive effect on metabolism and water potential of roots.

### EFFECTS OF LIGHT

Light has a direct effect on phloem loading through photosynthesis *via* the synthesis of sucrose and by providing energy. However, light also has an effect on the anatomy of the loading zone itself (Amiard et al., 2005, 2007). Depending on the loading mode (apoplastic or symplastic), the response to transfer from low light to high light (and therefore acclimation to increased photosynthesis) was different: in apoplastic species such as pea, cell-wall invaginations in the companion cells around the SE increased (Amiard et al., 2005). This indicated an increased exchange surface that allowed for higher sucrose phloem loading. On the contrary, in symplastic loaders such as pumpkin, plasmodesmatal frequencies did not increase, leading to starch accumulation in leaves (Amiard et al., 2005). The capacity of apoplastic loaders to increase the surface for membrane-mediated sucrose transfer around the conducting cells was further investigated (Amiard et al., 2007). Besides its role in nutrient exchange, cell-wall enlargement was proposed as protecting phloem cells against pathogens and insects.

### EFFECTS OF LOW TEMPERATURES

Low temperatures can affect phloem sugar transport in different ways, involving distinct cell types (intermediary cells, parenchyma transfer cells, SEs).Considering that species with a symplastic minor-vein configuration dominate in tropical regions and that species with an apoplastic configuration dominate in temperate zones, temperature is considered as a major parameter of the phloem-loading mode in plants. Symplastic loaders are considered as more cold-sensitive than apoplastic loaders (Gamalei, 1991; Van Bel and Gamalei, 1992). Gamalei et al. (1994) proposed that in herbaceous species and deciduous trees, the collapse of intermediary cells at low temperatures, which leads to decreased photo-assimilate loading, could explain the sensitivity of symplasmic phloem loading to cold. However, these ultrastructural changes have not been observed in broadleaf-evergreen species* (Ajuga reptans*, *Aucuba japonica*, and *Hedera helix*)**with a symplastic phloem-loading mode. The winter leaves of these plants have a higher exudation rate at low temperatures and no starch accumulation is observed in their chloroplasts. Therefore, the removal of excessive photo-assimilates from source leaves under low temperature may be necessary to maintain their functional and structural integrity and can thus be regarded as a result of cold acclimation (Hoffmann-Thoma et al., 2001). Later physiological studies (Schrier et al., 2000) revealed no differences between symplastic and apoplastic species in their response to cold. This led to the hypothesis that the phloem-loading mode was related to growth architecture rather than habitat, and was confirmed by a study by Davidson et al. (2011).

In monocot and dicot plant species, tocopherol (vitamin E) deficiency impairs photoassimilate export from source leaves *via* enhanced callose deposition in the vascular tissues (Hofius et al., 2004). The same effect has also been described for phloem loading under low temperature (Maeda et al., 2006). The *Arabidopsis* *vitamin* E2 (vte2) mutants, which lack α-tocopherol (the major tocopherol in leaves), exhibit dramatic phenotypes under low temperature. When they are transferred to non-freezing low-temperatures (+7.5°C), *vte2* mutants grow more slowly than wild-type plants, and accumulate significantly higher levels of anthocyanins. Accumulation of sucrose and other soluble sugars is much higher in *vte2* than in the wild-type after 60 h of low temperature treatment, although the photosynthesis and carbon fixation rates do not differ between the two genotypes. ^14^CO_2_-labeling experiments demonstrate that low-temperature-treated *vte2* plants translocate significantly less ^14^C-labeled photo-assimilates from leaves to roots. In *vte2* mutants, changes in cell structure occur exclusively within the phloem parenchyma “ transfer” cells, which exhibit irregular thickenings of cell-wall callose deposits. This process leads to a callose boundary between the phloem parenchyma “ transfer” cells and the SE/CC complex. Therefore, tocopherol prevents abnormal callose deposition in phloem parenchyma cell walls and thus maintains photo-assimilate transport at low temperature. In *Arabidopsis* plants acclimated to low temperature (+5°C), up-regulation of SUTs SUC1 and SUC2 expression represents another mechanism for maintaining sucrose transport to sinks (mainly young leaves; Lundmark et al., 2006).

In dicots, when short sections of stems or petioles are progressively exposed to cool temperatures (thermal jackets), phloem transport stops transiently through the cooled region (Faucher et al., 1982). This stoppage is local and transient as phloem transport can start again even if tissues are maintained at low temperatures (Peuke et al., 2006). Furthermore, the cooling rate determines stoppage duration. In fact, the effect of cooling depends on experimental conditions and SE structure. Conversely, in monocots, i.e., species that lack structural P-proteins (Dinant et al., 2003), assimilate translocation is not (maize) or moderately (wheat) affected by progressive cooling down to +1°C applied to leaves (Faucher and Bonnemain, 1981; Faucher et al., 1982). In maize, limited phloem transport still occurs after a progressive cooling down to -3°C, and ^14^C-assimilate allocation in the whole plant remains practically unchanged between +1°C and +40°C.

Further studies support the implication of sieve-element structural proteins in the cooling response (Lang and Minchin, 1986). The fact that lanthanum, a calcium channel blocker, can prevent the phloem response to cooling (Pickard and Minchin, 1990), strongly suggests that the response requires an increase in free Ca^2^^+^ in the SEs. This increase only occurs if the cooling process is rapid (White, 2009). Recent data indicate that forisomes (a class of P-proteins restricted to Fabacae), which control the immediate Ca^2^^+^-dependent occlusion of sieve tubes induced by injuries (Furch et al., 2007; Will et al., 2007), are also involved in the rapid transport blocking by cooling (Thorpe et al., 2010).

### EFFECTS OF CO_2_

The rise in carbon dioxide (CO_2_) in the atmosphere is suspected to be the main cause for global warming. Indeed, atmospheric CO_2_ concentration increased from around 315 ppm in 1959 to an average 390 ppm nowadays, and predictions give a CO_2_ concentration ranging between 540 and 970 ppm at the end of the century. This elevated atmospheric CO_2_ has a direct effect on plant photosynthesis: at the present atmospheric CO_2_ concentration, the photosynthetic reaction is limited by the low affinity of the active site of RuBisCO for CO_2_ in C3 plants (Drake et al., 1997; Woodward, 2002). An increase in CO_2_ should therefore enhance photosynthetic rates, carbohydrate production, and have a positive effect on phloem transport and growth. In fact, most of the plants grown in high CO_2_ effectively exhibit increased carbohydrate accumulation in leaves with biomass partitioning between source and sink organs differing according to species (Makino and Mae, 1999). Root/shoot ratios increase in herbaceous plants grown in high CO_2_ whereas they decrease in trees and remain stable in cereals (Farrar and Williams, 1991).

Classically, two high-CO_2_ acclimation steps are described, i.e., short-term and long-term acclimation (Drake et al., 1997; Cheng et al., 1998). Biomass formation is initially enhanced in the first days of exposure but this boosted growth is not sustained for a long time. The short-term response to high CO_2_ is an acclimation process whereby net photosynthesis, net carbon assimilation and growth are enhanced (Drake et al., 1997; Bae and Sicher, 2004). Excess sucrose is only partly exported to sink organs *via* the phloem and the resulting carbohydrate accumulation in leaves decreases the photosynthesis rate. A decreased RuBisCO content marks the beginning of long-term acclimation (Cheng et al., 1998; Bae and Sicher, 2004).

A comparison of sugar and starch contents in *Ricinus communis* leaves in plants grown at 350 or 700 ppm CO_2_ showed that leaves accumulated starch at 700 ppm. Starch accumulated because more sucrose was synthesized than consumed or exported to sink organs *via* the phloem (Grimmer et al., 1999). Phloem carbon export was induced by high CO_2_ at night. In the daytime, carbon export was independent of CO_2_ conditions, whereas at night the export rate dropped by 50% under normal CO_2_ but remained unchanged at high CO_2_. In fact, *R. communis* plants seemed to display sink limitation in the daytime and source limitation at night, and source limitation tended to be suppressed at high CO_2_ (Grimmer and Komor, 1999). In short, more sucrose was exported to sink organs at high CO_2_. The role of increased sucrose transport as a result of increased CO_2_ was also shown in *Arabidopsis thaliana* grown under 900 ppm CO_2_: the plants exhibited enhanced root growth, with increased root length, root diameter and root number, and a modified branching pattern (Lee-Ho et al., 2007). The same root changes were noted on plants grown at 360 ppm CO_2_ and supplied with exogenous sucrose, confirming the role of sucrose transported from the source.

In *Opuntia ficus-indica*, a CAM plant, no decrease in photosynthesis was detected in long-term exposure at high CO_2_. After three months of CO_2_ enrichment, cladodes displayed an increase in glucose, starch, and malate contents, but no change in their sucrose content was measured (Wang and Nobel, 1996). The sucrose content in mother cladodes was stable because it was exported to daughter cladodes by an enhanced phloem transport that resulted in a 73% increase in daughter cladode biomass after 3 months of exposure to high CO_2_ (Wang and Nobel, 1996). However, data analysis from different plant species grown under high CO_2_ shows that phloem loading cannot alone account for variations in shoot carbohydrate partitioning. Increased CO_2_ can also have negative effects on plants. Due to an imbalance in nitrate assimilation caused by high CO_2_, protein accumulation in wheat grains is low despite an unchanged yield (Pleijel and Uddling, 2012). However, this is not the case for woody plants, like pine trees, which preserve seed quality while increasing seed production (Way et al., 2010).

### EFFECTS OF SOME SOIL AND AIR POLLUTANTS

#### Effects of cadmium

Some pollutants like heavy metals, cadmium (Cd), lead (Pb), or mercury (Hg) and the metalloid arsenic (As) are present in soils all over the world. Concerning Cd mobility within the phloem and its impact on sugar transport, little information is available, due to technical hurdles regarding phloem sampling (Mendoza-Cozatl et al., 2011). However, a low-affinity Cd transporter, *OsLCT1*, involved in phloem loading and accumulation in seeds, was identified in rice (Uraguchi et al., 2011), but no study related to sugar transport was carried out. Another experiment was led on willows used for Cd phyto-extraction. In those trees, sieve tubes and companion cells degenerated in proportion to increasing Cd concentrations supplied at the root level (Vollenweider et al., 2006). Long-distance transport was therefore impaired and a reduction in leaf size and biomass was observed (Cosio et al., 2006). Phloem degeneration was also noticed on maize grown on a Cd-contaminated soil (Cunha et al., 2008). Moreover, in willows, phloem regeneration was hindered due to reduced cambial activity (Vollenweider et al., 2006).

#### Effects of ozone

Tropospheric ozone is the most widespread air pollutant in many areas of the industrialized world and the overall ozone concentration has increased over the past decades as a result of anthropogenic activities (Krupa and Manning, 1988; Volz and Kley, 1988). Ozone mainly originates from photochemical reactions of volatile organic compounds with nitrogen oxides (NOx) released from anthropogenic and natural sources (Stockwell et al., 1997). Ozone causes a series of negative effects on vegetation such as decreased photosynthesis and growth, enhanced premature senescence and reduced crop yield (Pell and Dann, 1991; Sandermann, 1996). O_3_ alters chloroplast membranes and decreases photosynthesis by reducing RuBisCO activity and concentration (Grams et al., 1999), which suggests that its main target is the photosynthetic apparatus (Kangasjärvi et al., 1994). Thus the availability of photo-assimilates for sink organs is decreased (Fiscus et al., 2005; Grantz et al., 2006).

Grantz and Yang (2000) tried to understand whether the impact of O_3_ on reduced carbon allocation in plants was due to source limitation or inhibition of translocation. The results indicate that ozone has direct effects on phloem transport with consequent inhibition of translocation to roots, as previously suggested by Mortensen and Engvild (1995). These data are consistent with a primary effect on phloem loading and secondary feedback inhibition of photosynthesis (Grantz, 2003; Grantz et al., 2006).To gain information on this phenomenon, a meta-analysis of all the data available about the impacts of O_3_ on root/shoot allocation and growth was performed by comparing RGR (relative growth rate of the whole plant) values and the allometric coefficient k (k = RGR_root_/RGR_shoot_). The results show that both parameters were significantly reduced by ozone but k showed more variability than RGR. This could indicate that root allocation is disturbed by O_3_ but photo-assimilate availability is not. This result is consistent with an inhibition of photo-assimilate translocation rather than with a limitation of the photosynthetic process (Grantz et al., 2006).

Carbon translocation from source leaves of Pima cotton has been directly studied by monitoring ^14^C-labeled photo-assimilates during a sudden exposure to O_3_. The results indicate that the total labeled carbohydrates transported from source leaves were reduced by O_3_ through effects on assimilation (up to 20%) and on export from leaves (up to 70%; Grantz, 2003; Grantz et al., 2006). Another study examined the translocation velocity of ^14^C-labeled photo-assimilates in wheat : although the authors observed no significant difference in the translocation velocity in O_3_-treated plants, the amount of carbon transported decreased (Mortensen and Engvild, 1995). In conclusion, O_3_ could induce changes in carbon allocation or partitioning probably due to decreased amounts of transported carbon. All those works highlight that the major impact of ozone is the reduction of phloem loading probably linked to oxidant damage on plasmalemma or plamodesmata in mesophyll or phloem companion cells (Grantz and Farrar, 1999).

O_3_ exposure could also have an indirect effect on plants by blocking phloem translocation *via* the induction of callose deposition on phloem sieve plates (Wilkinson et al., 2012). In potato, accumulation of callose in the phloem and starch in the parenchyma cells of source leaves was observed after ozone exposure. O_3_ also decreased tuber weight, supporting the hypothesis of impaired phloem functioning (Asensi-Fabado et al., 2010). A better understanding of the effects of O_3_ on carbohydrate translocation could come through the study of apoplastic and symplastic phloem-loading species to confirm the oxidant impact of O_3_ on membranes (Grantz and Yang, 2000).

#### Effects of sulfur dioxide

Sulfur dioxide (SO_2_) was a major air pollutant during the second half of the 20th century and was considered as the main cause of forest decline in central Europe (Kurczyńska, 1997). SO_2_ is highly soluble in water: a concentration of 0.035 ppm SO_2_ in the air can produce up to 35 ppm SO_2_ in aqueous solution (Puckett et al., 1973). The pollutant can accumulate in leaf tissues and cause disturbances in physiological mechanisms such as photosynthesis, respiration, transpiration (Saxe and Murali, 1989). In bean leaves (Minchin and Gould, 1986) and castor bean cotyledons (Lorenc-Plucinska and Ziegler, 1987), photo-assimilate translocation is also affected due to inhibited phloem loading, independently of reduced photosynthesis. In broad bean, H^+^/ATPase and SUTs have been identified as possible targets, both in leaf discs (Maurousset et al., 1992b) and plasma membrane vesicles (Maurousset et al., 1992a). More recently, an anatomical study showed that SO_2_ led to a decreased number of phloem cells, but it was difficult to discriminate between general reduction of cambial activity and disturbances in the division of phloem mother cells (Kurczyńska, 1997).

## EFFECTS OF BIOTIC STRESS

**FIGURE 3:**
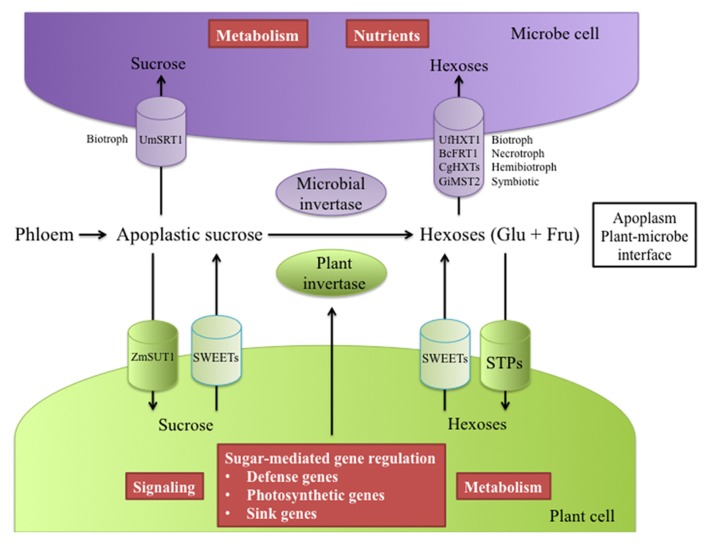
**Simplified representation of the key players involved in the competition for sugars at the plant/microbe interface.** Depending on the pathosystem, plants and microbes present efficient machineries to take up or modify apoplastic sucrose. In biotrophic interactions, sucrose can be taken up by both host and fungus *via* sucrose transporters, e.g., maize ZmSUT1 and fungus *Ustilago maydis* UmSRT1, respectively. However, glucose is the main carbon source transferred from the host to the parasite and is essential for the feeding and metabolism of the parasite. Cell wall invertases from host and microbes contribute to the source of hexoses at the apoplast level. Hexose transporters allow pathogenic or mutualistic fungi to preferentially compete for glucose and/or fructose (i.e., UfHXT1, BcFRT1, CgHXTs, GiMST2). To gain access to apoplastic hexoses, plants possess a large repertoire of STPs that can support host demand. Multiple roles of hexoses in host cells have been described; among others, hexoses can be used as an energy source or as signaling molecules and regulators of pathogenesis-related, photosynthetic and sink gene expression. An indirect consequence of host sucrose and hexose acquisition is a possible starvation of microbes through a limited access to sugar at the interface. Host sugar uptake can be bypassed in some pathogenic interactions. Specific effectors (not represented in the diagram) released by some bacteria and probably fungi can manipulate host sugar effluxers (SWEETs) and further make sucrose and hexoses available for the pathogen

During their development, plants have to deal with the presence of microbes, like fungi, viruses, bacteria and also herbivores and sometimes other plants that act as parasites. Those organisms, whatever their type, develop at the expense of the sugars produced by plants (**Figure 3**), and may therefore affect phloem transport of sugars.

### MUTUALISTIC AND PATHOGENIC MICROBES

Microorganisms can be separated into two groups according to their lifestyles, mutualistic (e.g., mycorrhiza) and pathogenic (biotrophic to necrotrophic; Newton et al., 2010). Even if their modes of colonization are different, microorganisms have evolved sophisticated strategies to avoid, suppress or bypass plant defenses and to divert nutrients, especially sugars, from the host plant for their growth (**Figure 3**). For example, mutualistic microorganisms and biotrophic pathogens can grow within the plant through complex interfaces, arbuscules and haustoria respectively, through which nutrients are transferred (Voegele and Mendgen, 2011; Smith and Smith, 2012). In contrast, necrotrophic pathogens secrete toxins and produce hydrolytic enzymes that kill host cells in order to feed on macerating tissues (van Kan, 2006).

Microbes can colonize either sink or source organs. Because both mutualistic and pathogenic interactions require sugar supply from host plants to the heterotrophic colonizing agent, they interfere with the source-sink balance. In most cases, it is largely assumed that colonized source organs are subjected to a source-to-sink transition that modifies the mechanism of sugar transport and partitioning at the whole plant level (Biemelt and Sonnewald, 2006). Among pathosystems, interactions between plants and biotrophic fungi are often cited as models for the study of pathogen-related modifications of carbon partitioning. For this reason, we particularly focus here on plant-biotrophic fungus interactions and only mention a few distinctive features of other pathosystems.

Biotrophic fungi, e.g., rust, powdery mildew, establish a long-term feeding relationship with the living cells of their hosts through the formation of haustoria. These are penetrating cell-wall structures that leave the protoplast of host cells intact and create an apoplastic interface through which released host nutrients are absorbed by the fungus (Mendgen and Hahn, 2002; Panstruga, 2003). Autoradiography studies using radiolabeled substances give indirect evidence for the central role of haustoria in sugar and amino-acid transfer from host to biotrophic pathogens (Hall and Williams, 2000; Voegele and Mendgen, 2011). In infected tissues, the fungal carbon demand creates an additional major sink that competes with host sinks. Competitiveness between plant and fungal sinks has been recently examined using a combined experimental-modeling approach. The authors showed that, in wheat infected by the leaf rust fungus *Puccinia triticina*, fungal sporulation had a competitive priority for photo-assimilates over grain filling (Bancal et al., 2012).

The nature of the host carbon energy source (hexoses or sucrose) transferred through the haustoria has been a matter of debate as to the origin of the apoplastic sugars taken up (**Figure 3**). Rather than sucrose, glucose appears to be the major carbohydrate imported from the host to the parasite, e.g., powdery mildew (Sutton et al., 1999; Hall and Williams, 2000). Apoplastic sucrose is most likely hydrolysed by cell-wall invertases (cwINV) which are key players in supplying carbohydrates to sink tissues (Roitsch and Gonzalez, 2004). Many studies report increased invertase activity in response to powdery mildew or other pathogens and in different plant species (Roitsch et al., 2003; Kocal et al., 2008; Siemens et al., 2011). This increase in cwINV activity in infected tissues constitutes a major driving force in sugar unloading. For most pathosystems, especially with obligate pathogens, it is difficult to discriminate between plant or pathogen contribution to the induced cwINV activity (**Figure 3**). While several plant cwINV genes, whose expression is correlated with increased cwINV activity, have been identified (Fotopoulos et al., 2003; Hayes et al., 2010), the pathogen’ s needs for carbohydrates are unlikely to be solely met by the enzymatic machinery of the host. So far few studies have reported fungal cwINV involved in such activities. The characterization of rust fungus *Uromyces fabae* Uf-INV1 suggests a fungal contribution to the higher cwINV activity in the biotrophic interaction with the host plant *Vicia faba* (Voegele et al., 2006). Regarding the necrotrophic parasite *Botrytis cinerea*, a contribution of the fungus to higher cwINV activity during infection of *Vitis vinifera *has been evidenced**(Ruiz and Ruffner, 2002). Accordingly, both partners appear to activate their own invertases, providing strong support to the theory that infection by pathogens creates a new sink that competes with existing sinks (**Figure 3**). As a consequence, hexoses accumulate in the apoplast, and are taken up by co-regulated hexose transporters (Wright et al., 1995; Clark and Hall, 1998).

High extracellular sugar levels are somehow beneficial for both partners. On the plant side, sugars act as signaling molecules that can regulate many physiological processes, including defense mechanisms through the control of gene expression (Herbers et al., 1996; Roitsch et al., 2003; Rolland et al., 2006; **Figure 3**). For example, sugars induce pathogenesis-related genes and repress photosynthetic genes (Roitsch, 1999; Bolouri Moghaddam and Van den Ende, 2012). An indirect host defense strategy consists in starving the pathogen by limiting host sugar availability at the interface. Reports describe an increased capacity for glucose retrieval by host tissues after challenge by biotrophic as well as necrotrophic pathogens (Fotopoulos et al., 2003; Azevedo et al., 2006). Some plant monosaccharide transporters (MSTs) are involved in sugar resorption upon infection (Buttner, 2010; Slewinski, 2011). This is exemplified by the report of the up-regulation of the plant cell-wall invertase *AtβFRUCT1* and the hexose transporter *AtSTP4* in *Erysiphe cichoracearum*-infected *Arabidopsis* leaves, which correlates with increased invertase activity and glucose resorption (Truernit et al., 1996; Fotopoulos et al., 2003). This supports the functional coordination of STPs and cwINVs in the supply of sink tissues with hexoses (Sutton et al., 2007; **Figure 3**). Further molecular evidence of a competition for apoplastic glucose has been provided in infected broad bean by the identification and characterization of rust *Uromyces fabae* sugar transporter UfHXT1, which is localized in the haustorial plasma membrane. UfHXT1 preferentially transports glucose and fructose rather than sucrose to the fungus (Voegele et al., 2001). Substrate specificity and localization of such fungal MSTs facilitates plant hexose assimilation and thus participates in fungal sink strength (**Figure 3**).

Mutualistic or pathogenic microorganisms use a wide range of different strategies to gain access to carbohydrates from host plants, as highlighted in **Figure 3**. Mycorrhizal fungus *Glomus *high-affinity MST2 has been identified as a major player in sugar uptake with a critical function in the establishment of symbiosis (Helber et al., 2011; Doidy et al., 2012). Five hexose transporters (CgHXT1-5) have been characterized in the maize hemibiotrophic pathogen *Colletotrichum graminicola*, with large substrate specificities. CgHXT genes are differentially expressed during all infection phases, whether biotrophic or necrotrophic (Lingner et al., 2011). A high-affinity fructose transporter (BcFRT1) has been found in the necrotrophic fungus *B. cinerea*. Roles for fructose as a potent inducer of fungus germination have been suggested (Doehlemann et al., 2005).

Sucrose is the main photo-assimilate translocated from source to sinks. Upon release from the phloem in sink organs, sucrose is unloaded into the apoplast and is potentially exploitable by the fungus. In infected tissues, apoplastic sucrose uptake by fungal cells is believed to require the presence of fungal SUTs localized in the haustorial structure. The identification of SRT1, a highly specific SUT from the corn smut fungus *Ustilago maydis*, suggests that this fungus can efficiently use apoplastic sucrose (Talbot, 2010; Wahl et al., 2010). *Ustilago maydis* hyphae grow along the phloem of infected maize plants where they have access to sucrose released from the phloem. Such a transporter (i.e., SRT1) allows the pathogen to compete for sucrose with sink cell sucrose transporters (SUC/SUT) at the plant/fungus interface (Wahl et al., 2010; Doidy et al., 2012). During the maize/*Ustilago maydis* interaction, competition for extracellular sucrose between the SUTs ZmSUT1 and UmSRT1 has been described, and SRT1 turned out to be essential for fungal virulence (Wippel et al., 2010). Direct sucrose uptake is probably an integral part of the pathogen’ s strategy to prevent plant defense responses being triggered by hexoses (mostly glucose) released from sucrose hydrolysis (Ehness et al., 1997). The identification and characterization of other fungal SUTs is not yet achieved and constitutes an open field to better understand the competition for sugars that takes place between the plant and the fungus (Doidy et al., 2012).

Recently, key insights into how microbes acquire the ability to use the host sugar efflux machinery for nutrient supply have been gained thanks to the discovery of a new class of plasma membrane-localized sugar transporters (**Figure 3**). Plant SWEETs function as facilitators of sugar influx and efflux. SWEETs were at first identified as glucose uniporters but paralogues (i.e., AtSWEET11 and AtSWEET12) can also export sucrose (Chen et al., 2010, 2012). Several SWEET genes are specifically regulated upon pathogen attack. Different patterns of expression have been reported after challenge by either bacterial (*Pseudomonas syringae* pv tomato strains) or fungal (the necrotroph *B. cinerea* or the biotroph *Golovinomyces cichoracearum*) pathogens. Authors also described a model in which OsSWEET11 and 14 expression is specifically targeted by *Xanthomonas oryzae* pv *oryzae* effectors to increase sugar efflux into the apoplast (Chen et al., 2010). Both specific bacterial effectors and OsSWEET expression are required for bacterial virulence, suggesting that pathogens probably take advantage of the SWEET-induced sugar efflux mechanism to gain access to sugars in cells around the infection site in order to support their own growth. The identification of this non-conventional family of sugar transporters highlights additional complexity and opens new perspectives onto our knowledge about sugar partitioning during plant-pathogen interactions.

### VIRUSES

Among plant pathogens, viruses are unique because they remain exclusively in the symplast of their host (Schoelz et al., 2011). This mode of colonization requires viruses to move from infection site to systemic tissues *via* the symplastic continuity created by cell-to-cell connections (plasmodesmata, PD) and the phloem long-distance translocation system (Lucas and Wolf, 1999; Gosalvez-Bernal et al., 2008). Viral infection involves virus-encoded movement proteins (MPs) which alter the exclusion size of PDs, suggesting that viruses can exploit the PD-mediated cell-to-cell trafficking of photo-assimilates. Carbohydrate allocation and signaling can be directly affected during virus infection. The mechanisms of these metabolic changes caused by viral infection have been assessed using transgenic expression of viral MPs; Olesinski et al., 1996; Hofius et al., 2001). Plants expressing viral MPs exhibited dilated PDs associated with significant physiological alterations such as changes in host primary metabolism, accumulation of starch and soluble sugars, decreased photosynthesis and increased respiration (Tecsi et al., 1996; Balachandran et al., 1997; Herbers et al., 2000). These changes strongly suggest that virus-infected leaves function as sinks. However, the effects of viral MPs on carbohydrate allocation can vary according to the way viruses exploit the host transport system. In some cases, it is not related to the PD size exclusion limit, but may rather be due to induced callose deposition at the PD level which consequently blocks symplastic sucrose transport (Biemelt and Sonnewald, 2006).

Virus-induced reallocation of host resources and its mechanisms seem to be virus-specific and result from interactions between specific viral and host components (Culver and Padmanabhan, 2007). For example, in Cucumber Mosaic Virus (CMV)-infected melon, modifications in phloem sap sugar composition, such as an increase in sucrose content, have been reported (Shalitin and Wolf, 2000). While cucurbits are known to be symplastic loaders, the identification of a SUT, CmSUT1, which catalyzes the active apoplastic loading of sucrose into the phloem of CMV-infected melon, provides evidence for a possible symplast/apoplast switch in sucrose loading (Gil et al., 2011).

### APHIDS

Aphids, which are the vectors of numerous plant viruses (Brault et al., 2010; Dedryver et al., 2010), are “ experts” at probing the phloem and at manipulating the plant tissues to their own advantage (Miles, 1999; Will et al., 2007). Using fine stylets, they drill into tissues intercellularly, making tiny punctures, and wait a few seconds to analyze the physicochemical properties of the microenvironment around the stylet tip (Tjallingii, 2006). Experiments using artificial systems indicate that the ability of aphids to find sieve tubes is linked to their ability to sense high sucrose concentrations and pH (Hewer et al., 2010).

Aphids constitute an additional sink that can modify assimilate allocation at the whole plant level, especially at the expense of the stem apex (Hawkins et al., 1987; Girousse et al., 2003). Data from various controlled infestations of alfalfa stems by pea aphids indicate that the reduction of the stem elongation rate (SER) is only partly explained by assimilate withdrawal and suggests that extra signals associated to pea aphid probing and feeding are involved in SER reduction (Girousse et al., 2003). In addition, dramatic changes in carbon and nitrogen allocation were observed under growth-chamber conditions using severe and short-time aphid infestations. They mainly consist in nitrogen mobilization from some parts of the stem, especially the apex, to the middle part of the zone of aphid infestation (Girousse et al., 2005). Complementary approaches show that aphid colonization induces changes in the expression of genes associated with sugar and nitrogen metabolism (Voelckel et al., 2004; Divol et al., 2005; de Vos et al., 2007) and causes an increase in the mRNA levels of a MST in infested tissues (Moran and Thompson, 2001). The systemic impact of aphid infestation also concerns source tissues (Miles, 1989). For instance, a single *Aphis fabae* on one side of a leaf grows faster if an *Aphis fabae* colony is feeding on the other side (Dixon and Wratten, 1971), suggesting the importance of aphid-related sugar accumulation. Aphid (*Myzus persicae*) infestation of *Arabidopsis* leaves leads to a dramatic increase in sucrose and starch contents in source tissues despite pest feeding (Singh et al., 2011). These changes suggest a stoppage of sugar export to the plant sinks. Infestation also induces an increase in trehalose levels. This change in trehalose metabolism promotes re-allocation of carbon into starch at the expense of sucrose, the primary energy source of the pest, and plant defenses *via* the induction of the PHYTOALEXIN-DEFICIENT4 gene (Singh et al., 2011). It is noteworthy that the trehalose found in the aphid hemolymph at millimolar concentrations as an energy source is also a plant signal that contributes to controlling infestation by phloem-sucking pests such as *M. persicae* at micromolar concentrations.

### PARASITIC PLANTS

Many plants like *Phelipanche* (Orobanche), *Cuscuta*, and *Striga* are able to establish parasitic relationships with a large number of crop plants, and this results in important productivity losses. Fighting against these parasitic plants is particularly complex because many treatments are also active on the host plants. Parasitic plants can be classified into two categories: hemiparasites are green, contain chlorophyll and can therefore have a photosynthetic activity. They take nutrients from the xylem sap in the wood of their host, can reduce nitrate but also use organic nitrogen found in the sap of their host. Holoparasitic plants are not photosynthetic and are thus heterotrophic for carbon and depend on their host for sugars, water, and minerals (Abbes et al., 2009a). Parasitic plants establish their connections with the host at the level of the sap-conducting tissues. Several years ago, sugar trafficking between host plant and parasite was clearly demonstrated using radiolabelled molecules. Although it is altogether agreed that the transfer of water and minerals between host and parasite xylem vessels does not require a membrane, the connections between host and parasite phloem vessels are more disputed. Initial histological studies showed that plasmodesmata were absent (Behnke et al., 1990), suggesting an apoplastic transfer of sugars and other molecules (Wolswinkel, 1974; Jeschke et al., 1994). However, using a GFP specifically expressed in companion cells (Haupt et al., 2001) or a fusion GFP-TMV (Tobacco Mosaic Virus) MP (Birschwilks et al., 2006), secondarily formed interspecific plasmodesmata were shown to be open and functional. In addition, ^3^H-sucrose, 5,6-carboxyfluorescein and viruses were translocated from the host to the parasite, giving unequivocal evidence for a symplastic transfer of solutes (Hibberd and Jeschke, 2001; Birschwilks et al., 2006).

Different enzyme activities are involved in the parasitic mechanism. *PrSUS1*, a sucrose synthase isolated from the parasitic plant *Phelipanche ramosa*, exhibits a spatial and temporal regulation**during the infection process (Péron et al., 2012). Expression is regulated by auxin from the host plant. The authors suggest that PrSUS1 is involved in cellulose synthesis during the secondary thickening of differentiating xylem elements in the tubercles (i.e., globular structures developed after parasite seed germination that carry numerous adventitious roots and whose apical bud produces a subterranean shoot) and in the adventitious roots of *P. ramosa. *Cellulose synthesis is probably crucial for the cell-wall integrity of both xylem and phloem tissues. Another enzyme activity acting as the driving force in many source/sink relationships is the invertase activity involved in the cleavage of sucrose into glucose and fructose. Transcripts of *PrSai1 *that encodes a soluble acid invertase and the corresponding enzyme activity were high in growing organs during parasite fixation. In addition, germinated seeds displayed enhanced cell-wall invertase activity (PrCWI), suggesting its contribution to the sink strength of infected roots during the subsequent step of root penetration (Draie et al., 2011).

*Orobanche* also accumulates high amounts of polyols like mannitol, and this decreases the osmotic potential below that of the host plant (Harloff and Wegmann, 1993; Abbes et al., 2009b). This mechanism is essential for increasing the parasite’ s sink strength. A mannose-6-phosphate reductase (M6PR), the key enzyme of the mannitol biosynthesis pathway, was identified and cloned from *Orobanche* (Delavault et al., 2002). Expression of that gene was induced at the attachment stage and sucrose from the phloem host was rapidly converted into mannitol. Consequences are increases in host nutrient uptake and in parasite protein synthesis and vegetative growth. Accumulation of mannitol in *Orobanche* tissues is strongly enhanced following the haustorial connection. Targeting the corresponding enzyme activity could be a major strategy for fighting against these parasites (Delavault et al., 2002).

All these relationships in terms of carbon and nitrogen exchanges are very important to establish susceptibility or tolerance to *Orobanche*. Phloem exudates of a *faba *bean tolerant line were highly deficient in nitrogen compared to those of the susceptible line (Abbes et al., 2009a). In addition, after haustorial development and phloem connections were established with the tolerant line, soluble invertase activity was very low. Taken together, these results indicate that the reduced growth of *Orobanche* on the tolerant *faba *bean line resulted from a reduced capacity to use the host-derived carbohydrates and lower sink strength.

Using radiolabelled valine and asparagine, amino acids were also shown to be transported from the host to *Cuscuta europaea* (Wolswinkel et al., 1984). More recently, Aly et al. (2011) demonstrated that several other molecules such as proteins and macromolecules could be translocated to *Phelipanche aegyptiaca* through phloem connections. These results suggest that targeting the delivery of proteins and/or nucleic acids could be very interesting in the development of parasite-resistant strategies. The use of herbicides could also be a strategy to get rid of parasitic plants. Glyphosate, a systemic herbicide, is accumulated in the parasite due to its strong sink activity, without significant damage to the host (Nadler-Hassar et al., 2004). Glyphosate usually inhibits 5-enolpyruvylshikimate-3-phosphate synthase (EPSPS), a key enzyme in the shikimate pathway. However, in parasitic plants, glyphosate also blocks the transfer of sugars and macromolecules to the parasite. Therefore its toxic effect is more due to the inhibition of phloem transport to the parasite than to the inhibition of aromatic amino acid synthesis.

## CONCLUSION AND PERSPECTIVES

Plant life cycle is characterized by source-sink transitions due to changes in sink strength or in the number of sink organs competing for a common pool of sugars (Roitsch, 1999). The phloem plays a major role in connecting source and sink organs and supplying sugars, mainly in the form of sucrose, to sinks. As demonstrated in this review, phloem transport of sugar is tightly regulated and is very sensitive to alterations in a plant’ s environment resulting in changes in carbon allocation to sinks. However, there are few reports on the effects of biotic and abiotic factors on phloem transport and dealing with all components from source to sink.

At the source level, sucrose availability for export is dependent on photosynthetic activity. Interestingly, in many cases of adverse environmental conditions when photosynthetic carbon fixation was reduced, photosynthesis was not the primary target of the stress. Phloem transport of sugars was also affected, earlier than photosynthesis, leading to an increase of sucrose concentration in leaves and a feed-back inhibition of photosynthesis and sucrose export (Ainsworth and Bush, 2011).

Recent works have pointed out sucrose concentrations in the cytosol of mesophyll cells as a key factor for the regulation of sucrose export. The characterization of tonoplastic hexose and SUTs provides new support for the role of transient sugar storage in the vacuole to control the cytoplasmic sucrose concentration (Wingenter et al., 2010).

One interesting conclusion is that the structure of phloem cells can be altered by several abiotic stresses (light, SO_2_, O_3_). As the ultra-structure and integrity of such cells are poorly investigated, these alterations may be more frequent than reported. Additional evidence for the plasticity of phloem companion or parenchyma cell-wall comes from several studies on the effect of light (Amiard et al., 2005, 2007). Structural changes in the cell-wall and invaginations of the plasma membrane can increase fluxes of assimilates in the case of high light. Conversely, there are several reports of callose synthesis at different levels in the SEs, in response to external biotic or abiotic stresses. This physical constraint leads also to impaired transport of sucrose to sinks.

At the sink level, environmental cues can alter priority between different sinks: for example, increase in the root to shoot ratio is induced by mineral deficiency and both sucrose and ions are signals between root and shoot. Stress-related increase in sucrose or polyol delivery to sinks (e.g; roots but also seeds) is important for sink growth, cell turgor, and water potential maintenance.

Little is known about the regulation of sugar transporters during abiotic stress at the molecular level despite their important role in the allocation of sugars in plants. This knowledge gap is due to the fact that many studies were conducted at a physiological level. Understanding the changes in transporter expression during stress is therefore a major challenge in order to predict and act on plant responses. Interesting clues on possible regulation by environmental and biotic factors were obtained by searching for putative regulatory elements in the promoters of sugar transporters in grape and *Arabidopsis* (Afoufa-Bastien et al., 2010). Recent data on biotic stress have unraveled the pathways of sugar exchange, and the corresponding molecular players have been identified (Doidy et al., 2012). Unexpectedly, sucrose can be absorbed by both host and pathogen, while it is commonly admitted that glucose resulting from the invertase activity is the main host sugar taken up by pathogens. These new findings highlight the complexity of sugar exchanges at the host/pathogen interface.

In order to understand further the distribution of carbon between sinks, future studies need to concentrate on the measurement of the phloem sap flux in relation with the expression of sugar transporters, taking advantage of new imaging techniques (Knoblauch and Oparka, 2012) combined with traditional ^14^CO_2_ labeling. Key information should be obtained from metabolomic approaches of phloem sap composition (Fiehn, 2003). As pointed out earlier in this review and by Ainsworth and Bush (2011), there are interesting possibilities for increasing source activity and sink demand by manipulating the expression of selected transporter genes (Schroeder et al., 2013).

Concerning the acclimation of plants to some major environmental adverse conditions (drought and salt stress, pathogens), several sugars transported on long-distances such as polyols can also be targeted for improving stress resistance (Merchant and Richter, 2011). Nevertheless, the role of other sugars such as trehalose as signaling molecules will have to be taken into account. There are still considerable efforts to be made before getting a clear understanding of the role of phloem transport on source-sink relationships under stress conditions, but any progress should have beneficial effects on crop production.

## Conflict of Interest Statement

The authors declare that the research was conducted in the absence of any commercial or financial relationships that could be construed as a potential conflict of interest.
